# Correction of self-reported BMI based on objective measurements: a Belgian experience

**DOI:** 10.1186/s13690-018-0255-7

**Published:** 2018-02-05

**Authors:** S. Drieskens, S. Demarest, S. Bel, K. De Ridder, J. Tafforeau

**Affiliations:** Department of Public Health and Surveillance, Scientific Institute of Public Health, 14, Juliette Wytsmanstraat, 1050 Brussels, Belgium

**Keywords:** Body mass index, Self-reporting, Validity, Misclassification, Correction

## Abstract

**Background:**

Based on successive Health Interview Surveys (HIS), it has been demonstrated that also in Belgium obesity, measured by means of a self-reported body mass index (BMI in kg/m^2^), is a growing public health problem that needs to be monitored as accurately as possible. Studies have shown that a self-reported BMI can be biased. Consequently, if the aim is to rely on a self-reported BMI, adjustment is recommended. Data on measured and self-reported BMI, derived from the Belgian Food Consumption Survey (FCS) 2014 offers the opportunity to do so.

**Methods:**

The HIS and FCS are cross-sectional surveys based on representative population samples. This study focused on adults aged 18–64 years (sample HIS = 6545 and FCS = 1213). Measured and self-reported BMI collected in FCS were used to assess possible misreporting. Using FCS data, correction factors (measured BMI/self-reported BMI) were calculated in function of a combination of background variables (region, gender, educational level and age group). Individual self-reported BMI of the HIS 2013 were then multiplied with the corresponding correction factors to produce a corrected BMI-classification.

**Results:**

When compared with the measured BMI, the self-reported BMI in the FCS was underestimated (mean 0.97 kg/m^2^). 28% of the obese people underestimated their BMI. After applying the correction factors, the prevalence of obesity based on HIS data significantly increased (from 13% based on the original HIS data to 17% based on the corrected HIS data) and approximated the measured one derived from the FCS data.

**Conclusions:**

Since self-reported calculations of BMI are underestimated, it is recommended to adjust them to obtain accurate estimates which are important for decision making.

## Background

Obesity is major public health problem [1–4]. Epidemiological studies have shown that a body mass index (BMI, the most commonly used indicator for relative weight among adults [5]) of 25–30 kg/m^2^ increases the risk of morbidity (cardiovascular diseases, type 2 diabetes and some types of cancer) and mortality [3, 4, 6]. A BMI of 30 or higher will further increase these risks [7]. In light of this growing problem, it is necessary to measure and monitor the prevalence of obesity in the general population as accurate as possible [4]. According to the national Health Interview Survey (HIS) of 2013 14% of the Belgian population can be considered as obese. Moreover, since the first survey in 1997, this proportion has increased with 27% [8].

The BMI calculated in the HIS is based on self-reported height and weight collected by means of a questionnaire. Such an approach is commonly used in large epidemiological studies [9–12] because collecting self-reported data is more feasible and less expensive than collecting objective measurements [2, 13–15]. Nevertheless, the inaccuracy of self-reported data has been well investigated. Generally, participants tend to overestimate their height and to underestimate their weight, in particular those being overweight or obese, resulting in an underestimation of their actual BMI [6, 7, 16–19]. Consequently, those individuals are misclassified into a lower BMI-category which leads to an underestimation of the prevalence of obesity in the population [3, 4, 13, 14, 20, 21]. Social desirability can largely explain this phenomenon and some subpopulation groups (women, youngsters and high educated people) are more prone to it [6, 7, 13, 15, 16, 18, 22]. In Belgium, ‘prevention and health promotion’ is organised at regional level. For policy decisions and prevention programmes, especially in high-risk subpopulations, it is crucial to obtain BMI estimates that are as accurate as possible in order to draw reliable conclusions [23, 24]. If the aim is to continue to rely on self-reported HIS data, it is thus recommended to adjust those estimates so that they approximate measured data [21, 25].

In 2014, a national Food Consumption Survey (FCS) was conducted in Belgium. Both measured and self-reported body height and weight were collected, making it possible to study potential differences between measured and self-reported BMI, and accordingly to estimate the degree of BMI-misclassification. This was an opportunity to investigate reporting bias at national level in Belgium.

The objective of this study is to calculate correction factors based on FCS data by comparing the self-reported and measured BMI and to apply these factors to the self-reported BMI of the HIS. Although studies have stated that correction equations should not be applied across datasets [2, 17, 18], our study assumes that it is feasible in a certain context (e.g. same time span, similar target population and equivalent sampling method). We also assume that the corrected self-reported BMI of the HIS will be more valid, resulting in a more accurate BMI-classification.

## Methods

### Survey methodology

This study focused on adults aged 18–64 years, since the FCS targeted the Belgian population of 3–64 years and the relative weight of children and youngsters is not yet stable [26]. The HIS and the FCS are both cross-sectional surveys. The last HIS was conducted in 2013, the last FCS in 2014. A sample of the population was selected, targeting all persons residing in Belgium without restriction on their place of birth, nationality or other characteristics. Both surveys used quarterly updates of the National Population Registry as sample frame. A multistage clustered sample design was applied in both surveys involving a geographical stratification, a selection of municipalities within the provinces and of respondents within municipalities. The difference between the two surveys was that in the HIS the respondents were selected at household level (maximum 4 persons per household) and in the FCS at individual level. The use of matched substitution of non-participating respondents/households ensured the realisation of the predefined net-sample size and composition. Proxies were allowed in both studies. The methodology of the HIS has been described by Demarest et al. [27] and that of the FCS by Bel et al. [28]. Both surveys were carried out in line with the Belgian privacy legislation and approved by the ethical committee of Ghent University.

### Study populations

In the HIS, a total of 10,829 citizens was interviewed, 6747 of them belonging to the age group 18–64 years. The overall participation rate at household level was 57%. Self-reported body height and weight were collected using a Computer Assisted Personal face-to-face Interview (CAPI) at the participant’s home. The following questions were asked: ‘How tall are you without clothes and shoes? (cm)’ and ‘How much do you weight without clothes? (kg)’. Pregnant women were asked to report their weight before pregnancy. Cases with a missing or invalid height and/or weight were excluded from the analysis (pregnant women could not be excluded). The final HIS sample contained 6545 individuals.

The participation rate of the FCS was 37%. Overall 3297 citizens participated, of which 1270 in the age group 18–64 years. This survey collected both self-reported and measured body height (in cm) and weight (in kg) for the same individuals using a CAPI, also at their home. Trained dieticians were used as interviewers and to gather the measured data. During the first 24-h food recall interview, body height and weight were self-reported. Participants were informed that their height and weight would be measured during the second home visit. The time lapse between the first and the second home visit was minimal 2 and maximal 4 weeks. During the second home visit, the anthropometric measurements were taken following a standardized protocol. The respondents were measured with light clothes and without shoes. Height was accurately measured to 0.5 cm using a stadiometer (type SECA 213 (Seca gmbh & co. kg, Hamburg, Germany)) and weight to 0.1 kg using an electronic scale (type SECA 815 and 804 (Seca gmbh & co. kg, Hamburg, Germany)). After excluding pregnant women and cases with a missing or invalid self-reported/measured height and/or weight, the study sample comprised 1213 individuals.

### Background variables

Studies have demonstrated that demographic, cultural and social characteristics of a population can influence the accuracy of self-reported data [3, 4, 7, 13, 15, 22]. Therefore the analyses also took into account, in both the HIS and the FCS, the following background variables: region of residency, gender, educational level, and age group. The educational level is based on the International Standard Classification of Education (ISCED) whereby the low educated people have at most a higher secondary education and the high educated people at least a post-secondary or tertiary education. A comparison was made of the distribution of the participants by background variables according to the study sample (HIS 2013 versus FCS 2014).

### Misreporting of the self-reported BMI in the FCS

The FCS dataset contains both measured and self-reported BMI, calculated respectively from the height and weight. The magnitude of misreporting of the BMI at population level was estimated. This was expressed in terms of the absolute difference, calculated as the mean measured BMI minus the mean self-reported BMI (negative in case of over-reporting and positive in case of under-reporting), and in terms of the relative difference, calculated as the mean measured BMI divided by the mean self-reported BMI. These calculations were stratified by the combination of four background variables: region (3) * gender (2) * educational level (2) * age group (3), resulting in 36 strata.

Misreporting of the mean BMI consequently lead to misclassification. According to the criteria of the World Health Organization (WHO), participants were categorized as underweight (BMI < 18.50), normal weight (BMI 18.50–24.99), overweight (BMI 25.00–29.99) or obese (BMI ≥ 30.00) [29]. The validity of the self-reported BMI-classification was evaluated by cross-tabulating the measured BMI-categories with the self-reported BMI-categories. The sensitivity and specificity of the obesity class was also assessed.

### Correcting the self-reported BMI in the HIS

Giacchi et al. [30] proposed a simple and economical procedure for adjusting the bias in the self-reported BMI. This procedure was applied to adjust the self-reported BMI of the HIS. Based on the FCS, a correction factor by stratum was calculated as the ratio between the measured and the self-reported BMI (the relative difference described earlier). Then, this correction factor was multiplied with the individual self-reported BMI of the HIS. In this way, a corrected BMI was produced for the HIS for the specific strata (region * gender * educational level * age group). To avoid having small numbers by strata, the categories by background variable were rather large. Producing a corrected BMI based on a corrected height and a corrected weight is very similar to a directly corrected BMI (used in this study). Both methods can be applied [20].

A Bland Altman plot analysis [31] was used to quantify the agreement between the measured BMI and the self-reported BMI of the FCS. Potential variation was assessed by the mean difference (đ) and the standard deviation (s) of the differences: đ ± 2 s, referring to the limits of agreement. A comparison was made with a Bland Altman plot between the measured BMI and the corrected self-reported BMI of the FCS (calculated in a similar way as the corrected BMI of the HIS). An improvement of the variation will be an argument for applying this correction factor on the HIS data.

Based on these corrected BMI’s, a new BMI-classification was generated for the HIS. The prevalence of obesity was then aggregated by background variable. The significant difference (based on the 95% confidence interval (CI)) was assessed between the obesity prevalence estimated with the corrected self-reported BMI of the HIS and the prevalence based on the measured BMI of the FCS.

All the analyses were performed with SAS® 9.2 [32]. For calculating the mean (PROC SURVEYMEANS) and the prevalence (PROC SURVEYFREQ) the complex survey design (weighting, clustering, and stratification) was taken into account.

## Results

### Distribution of the study samples by background variables

When comparing the distribution of the two study samples by different background variables (Table 1), it is most important to mention that in the HIS, the Brussels Region was oversampled, while in the FCS such oversampling was not foreseen.

**Table 1:** Distribution (number and proportion) of the study samples by background variables

		HIS 2013		FCS 2014	
Background variables		N	%	N	%
Region of residency	Flemish Region	2113	32.3	702	57.9
	Brussels Region	1911	29.2	90	7.4
	Walloon Region	2521	38.5	421	34.7
Gender	Males	3188	48.7	596	49.1
	Females	3357	51.3	617	50.9
Education	Low level	3503	52.5	556	45.8
	High level	3042	46.5	657	54.2
Age group	18–34 years	2155	32.9	456	37.6
	35–50 years	2397	36.6	414	34.1
	51–64 years	1993	30.5	343	28.3
Total		6545	100.0	1213	100.0

### Misreporting of the self-reported BMI in the FCS

Regarding the absolute differences, the mean self-reported BMI was significantly underestimated with almost one unit (0.96 kg/m^2^) when compared with the mean measured BMI (only 3% of the strata overestimated their self-reported BMI: males of 51–64 years in the Brussels Region with a low and high education level). Misreporting, expressed in absolute and relative differences, of the mean BMI by strata is presented in Table 2.

**Table 2:** Misreporting of the mean self-reported BMI by strata, FCS 2014

Region	Gender	Education	Age group	Mean measured BMI	Mean self-reported BMI	Abs. diff. ^a^	Rel. diff.^b^
Total	Total	Total	Total	26.17 (25.79–26.55)	25.20 (24.85–25.56)	0.96 (0.87–1.06)	1.038 (1.034–1.042)
Flemish Region	Males	Low level	18–34	24.41 (22.96–25.85)	24.21 (22.75–25.66)	0.20 (− 0.08–0.48)	1.009 (0.996–1.022)
			35–50	27.41 (25.63–29.18)	26.40 (24.85–27.96)	1.00 (0.26–0.48)	1.038 (1.018–1.057)
			51–64	28.82 (27.68–29.96)	27.94 (26.85–29.03)	0.88 (0.43–1.33)	1.032 (1.017–1.048)
		High level	18–34	23.79 (23.08–24.50)	23.07 (22.41–23.72)	0.73 (0.44–1.01)	1.031 (0.019–1.044)
			35–50	26.28 (25.30–27.27)	25.60 (24.69–26.52)	0.68 (0.49–0.88)	1.027 (1.019–1.034)
			51–64	26.20 (25.15–27.25)	25.35 (24.33–26.37)	0.85 (0.50–1.21)	1.034 (1.019–1.049)
	Females	Low level	18–34	26.02 (23.79–28.25)	24.60 (22.88–26.32)	1.42 (0.50–2.33)	1.056 (1.024–1.088)
			35–50	26.58 (24.33–28.84)	25.26 (22.99–27.52)	1.33 (0.76–1.90)	1.056 (1.034–1.079)
			51–64	28.51 (25.92–31.10)	27.00 (24.52–29.48)	1.51 (1.10–1.92)	1.057 (1.041–1.073)
		High level	18–34	22.87 (21.98–23.76)	22.10 (21.29–22.91)	0.77 (0.37–1.17)	1.034 (1.017–1.052)
			35–50	24.91 (23.84–25.97)	23.98 (23.02–24.94)	0.92 (0.63–1.22)	1.038 (1.026–1.050)
			51–64	25.67 (23.96–27.39)	24.51 (23.06–25.95)	1.16 (0.73–1.60)	1.045 (1.030–1.060)
Brussels Region	Males	Low level	18–34	25.60 (21.21–29.99)	24.93 (21.65–28.20)	0.67 (− 1.22–2.57)	1.025 (0.952–1.098)
			35–50	25.04 (20.39–29.68)	24.16 (11.40–36.92)	0.88 (− 7.24–8.99)	1.038 (0.691–1.384)
			51–64	25.03 (19.38–30.68)	25.19 (20.53–29.85)	−0.16 (− 1.30–0.97)	0.992 (0.948–1.036)
		High level	18–34	24.30 (22.03–26.57)	22.96 (21.78–24.15)	1.34 (− 0.31–2.98)	1.057 (0.986–1.128)
			35–50	25.39 (24.05–26.54)	24.62 (23.82–25.42)	0.67 (−0.22–1.57)	1.028 (0.993–1.063)
			51–64	23.99 (19.25–28.74)	24.19 (20.48–27.91)	−0.20 (− 1.75–1.35)	0.987 (0.913–1.061)
	Females	Low level	18–34	25.67 (18.97–32.37)	24.98 (17.75–32.21)	0.69 (− 0.20–1.58)	1.033 (0.987–1.079)
			35–50	33.54 (6.95–60.14)	32.17 (6.71–57.63)	1.38 (−0.58–3.34)	1.043 (0.976–1.110)
			51–64	27.35 (17.42–37.28)	25.60 (17.73–33.46)	1.75 (− 1.02–4.53)	1.062 (0.978–1.147)
		High level	18–34	22.87 (18.24–27.51)	22.03 (18.36–25.70)	0.85 (−0.23–1.92)	1.033 (1.000–1.066)
			35–50	25.64 (20.44–30.84)	24.13 (19.77–28.48)	1.51 (0.40–2.62)	1.056 (1.019–1.092)
			51–64	25.66 (19.95–31.36)	24.63 (19.23–30.04)	1.02 (−0.24–2.29)	1.041 (0.987–1.094)
Walloon Region	Males	Low level	18–34	24.93 (23.71–26.16)	24.37 (23.31–25.43)	0.56 (0.16–0.96)	1.022 (1.007–1.038)
			35–50	28.73 (26.21–31.25)	27.44 (24.78–30.11)	1.28 (0.83–1.73)	1.051 (1.029–1.072)
			51–64	28.02 (26.80–29.25)	26.89 (25.80–27.98)	1.14 (0.62–1.65)	1.043 (1.023–1.062)
		High level	18–34	25.28 (23.37–27.19)	24.69 (22.84–26.53)	0.59 (0.32–0.86)	1.022 (1.012–1.033)
			35–50	28.49 (25.85–31.12)	27.70 (25.14–30.26)	0.79 (0.55–1.02)	1.029 (1.020–1.037)
			51–64	29.85 (27.21–32.49)	28.33 (25.95–30.70)	1.52 (0.84–2.20)	1.053 (1.033–1.073)
	Females	Low level	18–34	25.00 (22.40–27.60)	24.19 (21.74–26.63)	0.81 (0.31–1.32)	1.034 (1.012–1.056)
			35–50	27.62 (25.37–29.88)	26.30 (24.02–28.58)	1.32 (0.52–2.12)	1.051 (1.022–1.081)
			51–64	27.50 (25.97–29.03)	26.18 (24.58–27.78)	1.32 (0.62–2.02)	1.053 (1.020–1.085)
		High level	18–34	24.59 (21.06–28.13)	23.60 (20.14–27.06)	0.99 (0.34–1.65)	1.040 (1.017–1.063)
			35–50	25.49 (23.30–27.67)	24.25 (22.26–26.25)	1.23 (0.81–1.65)	1.050 (1.034–1.066)
			51–64	27.71 (25.65–29.77)	26.61 (24.38–28.83)	1.10 (0.63–1.58)	1.043 (1.025–1.061)

^a^Absolute difference: mean measured BMI – mean self-reported BMI

^b^Relative difference: mean measured BMI / mean self-reported BMI

The overall misclassification was 16.2%. Among the obese people, 26.5% reported themselves as overweight and 1.3% as normal weight. The sensitivity of self-reported information on obesity was 72.2% and the specificity was 99.6%.

### Correction of the self-reported BMI in the HIS

The Bland Altman plot analysis indicates that the 95% limits of agreement between the measured BMI and the self-reported BMI of the FCS ranged from − 2.18 to 4.10. After correcting the self-reported BMI, this range has changed to − 3.09 to 3.10, indicating a more homogenous variation. The underestimation of the self-reported BMI has decreased which will improve the BMI-classification. This positive impact is an argument to also apply this correction factor on the HIS data (Fig. 1).

**Fig. 1:**
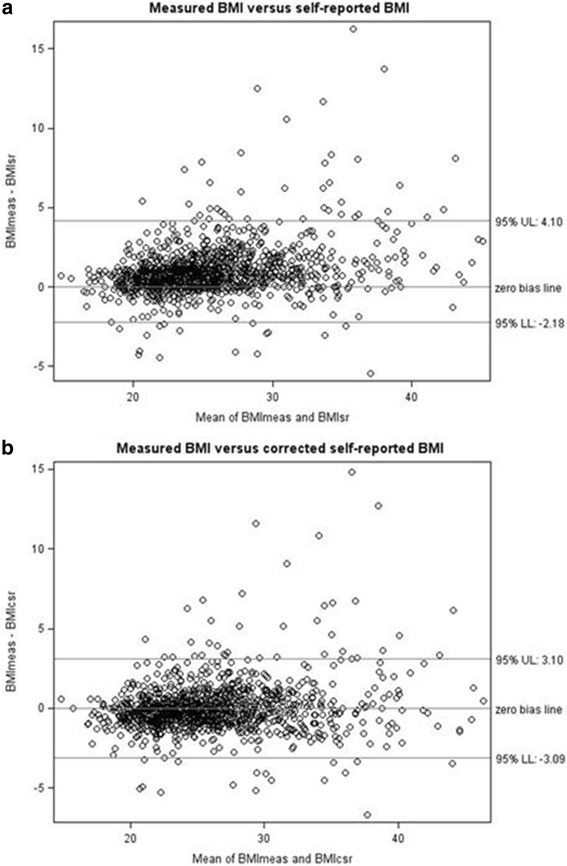
New proposition: Quantification of the agreement between the measured BMI and the self-reported BMI (**a**) compared to the measured BMI and the corrected self-reported BMI (**b**), Bland-Altman plots, FCS 2014

The prevalence of obesity according to the measured and self-reported BMI of the FCS versus the self-reported and corrected BMI of the HIS by background variables is presented in Table 3. According to the measured FCS data, 19.4% (16.6%–22.2%) of the Belgian adult population aged 18–64 years was obese, a figure significantly higher in comparison with the HIS results (12.8% (11.6%–14.0%)). When looking at the corrected estimate, the HIS obesity prevalence increased to 17.4% (16.1%–18.8%) which was no longer significantly different from the FCS measured one.

**Table 3:** Obesity prevalence (and 95% CI) according to the measured and self-reported BMI of the FCS versus the self-reported and corrected BMI of the HIS by background variables

Background variables		FCS measured	FCS self-reported	HIS self-reported	HIS corrected
	Total	19.4 (16.6–22.2)	14.1 (11.7–16.5) ^a^	12.8 (11.6–14.0) ^a^	17.4 (16.1–18.8)
Region	Flemish Region	18.2 (14.8–21.6)	11.3 (8.6–13.9) ^a^	11.4 (9.6–13.2) ^a^	16.1 (14.1–18.2)
	Brussels Region	14.1 (4.7–23.5)	13.4 (4.1–22.1)	12.1 (10.3–13.8)	15.2 (13.2–17.1)
	Walloon Region	23.5 (18.0–28.9)	19.7 (14.6–24.8)	15.5 (13.5–17.4) ^a^	20.4 (18.3–22.6)
Gender	Males	18.6 (14.8–22.3)	14.0 (10.7–17.3)	12.7 (11.0–14.5) ^a^	15.9 (14.0–17.8)
	Females	20.3 (16.1–24.4)	14.2 (10.6–17.8)	12.9 (11.3–14.4) ^a^	18.9 (17.0–20.8)
Education	Low level	26.8 (22.0–31.5)	19.0 (14.9–23.1)	17.1 (15.3–18.9) ^a^	22.1 (20.1–24.0)
	High level	13.8 (10.5–17.1)	10.4 (7.5–13.2)	7.9 (6.4–9.5) ^a^	12.2 (10.3–14.0)
Age group	18–34 years	12.4 (8.3–16.6)	9.2 (5.5–13.0)	7.8 (5.9–9.6)	10.1 (8.1–12.1)
	35–50 years	20.8 (16.0–25.5)	17.6 (13.0–22.1)	13.3 (11.3–15.3) ^a^	18.2 (16.0–20.4)
	51–64 years	25.0 (19.4–30.6)	14.9 (10.7–19.1) ^a^	17.8 (15.5–20.2)	24.6 (21.9–27.3)

^a^Significantly different from FCS measured

The self-reported obesity prevalence of the HIS was significantly different from the FCS measured one in the Flemish Region (11.4% (9.6%–13.2%) versus 18.2% (14.8%–21.6%)) and the Walloon Region (15.5% (13.5%–17.4%) versus 23.5% (18.0%–28.9%)). The corresponding corrected prevalences are no longer significantly different (respectively 16.1% (14.1%–18.2%) and 20.4% (18.3%–22.6%)). Also the corrected obesity prevalence for both men and women (respectively 15.9% (14.0%–17.8%) and 18.9% (17.0%–20.8%)) approximated the measured obesity prevalence (respectively 18.6% (14.8%–22.3%) and 20.3% (16.1%–24.4%)). Furthermore, an increase of the self-reported obesity prevalence of the HIS was observed after correction for both educational levels: from 17.1% (15.3%–18.9%) to 22.1% (20.1%–24.0%) for the low educated people and from 7.9% (6.4%–9.5%) to 12.2% (10.3%–14.0%) for the high educated people. These corrected self-reported prevalences were closer to the FCS measured ones (respectively 26.8% (22.0%–31.5%) and 13.8% (10.5%–17.1%)), whereby also the significant differences disappeared. Finally, the self-reported obesity prevalence in the age group 35–50 years (13.3% (11.3%–15.3%)) was underestimated and was significantly different from the measured obesity prevalence (20.8% (16.0%–25.5%)). After correction, the self-reported obesity prevalence in the HIS increased to 18.2% (16.0%–20.4%) and the differences were no longer significant with the measured FCS estimates.

## Discussion

The BMI measured in the FCS 2014 served as golden standard. Overall, the self-reported BMI was underestimated in the FCS 2014. The underestimation with one BMI-unit is within the range of other studies [16, 24]. Hence, the prevalence of obesity was underestimated when based on self-reported BMI which is in accordance with many other studies as well [3, 13, 17–19]. The misclassification frequency is an appropriate way to assess the accuracy of self-reported BMI [24]. Especially obese people had the tendency to underestimate their BMI. As in other studies, a very high specificity was observed for obesity (5;23;25). However, the value for the sensitivity was lower, in line with other studies (5;9;25).

Data from the FCS lend itself to estimate a simple correction factor (measured BMI/self-reported BMI) which improves the accuracy of the self-reported BMI. Since the FCS and the HIS were conducted in comparable conditions (same time span, target population and sampling method), this correction factor could be applied to the individual self-reported BMI of the HIS, the second objective of this study. Other studies affirm that external applicability of a correction factor can be done under certain conditions [17, 24, 33].

Via this correction procedure, the ultimate goal of this study, to improve the accuracy of the self-reported BMI-classification in the HIS, was achieved. The corrected obesity prevalence of the HIS (17.2%) approximated the one of the golden standard (19.4%). This implies that the problem of obesity in Belgium is 4% points higher than initially thought based on self-reported HIS data. The significant differences between the corrected obesity prevalence and the golden standard also disappeared after correction for the following subgroups: the Flemish and the Walloon Region, both genders, the low educated people as well as the high educated people and the age group 35–50 years.

Because of some shortcomings of this study, the prevalence of obesity could possibly be higher. First of all, the participants of the FCS knew indeed when responding to the questions about their height and weight, that they would be measured and weighed at a later stage. Reporting under such circumstances presumably lead to more truthful data [17, 34]. This effect could be even strengthened by the fact that the FCS is a specific nutrition survey by a professional dietician (versus a general health survey by an interviewer). In this case the correction factor is probably underestimated [2, 14]. Second, the participation rate of both surveys was rather small, especially for the FCS (37%). The low participation rate of the FCS can be explained by the context of the survey: the HIS is a general health survey, but the focus of the FCS is on nutrition and the participation to this survey is more intensive (two visits, a food diary, measurements). Moreover, it has been shown that people who refuse to participate are more often obese, which could also bias the estimates [19, 25]. Since participation to both surveys is not mandatory, it would be desirable to develop strategies to improve the response rate among the population [20]. Some other limitations of this study are the fact that the questions used to assess height and weight in the FCS were less clearly defined and could therefore be slightly different from the questions used in the HIS, and the fact that the selection of the respondents at household level in the HIS may introduce some clustering in the results on BMI. Finally, the distribution of the two samples by region does not completely correspond, especially for the Brussels Region. The smaller strata in the FCS for this region lead to bigger confidence intervals and probably to less accurate estimates of the correction factor.

The results demonstrate that caution is needed when interpreting the obesity prevalence deduced from self-reported height and weight. Underestimation of the obesity prevalence gives a distorted image of the real health burden, which is problematic for policy making [2, 15, 22, 24]. Although preference is given to measured height and weight for assessing the obesity prevalence accurately, it is not always possible to collect such data because of practical and budgetary reasons, especially in large and recurrent population surveys [17, 35]. Therefore, height and weight collected through interview remains an essential tool [22, 28, 35, 36]. However, in this situation it is worth applying a correction factor to the self-reported BMI in order to increase the accuracy of the information and obtain more reliable estimates of the obesity prevalence. Since certain subgroups have a bigger influence on misreporting then others, it is important to determine this correction factor by specific background variables.

Other studies also recommend adjustment of self-reported data as a reasonable alternative when measurements are not feasible [3, 15, 20, 21, 25, 35–37]. Nevertheless, the correction factors of the FCS 2014 will likely not be applicable to the self-reported data of the forthcoming HIS’s since studies have indicated that reporting bias may change over time and should therefore be updated regularly [3, 17, 20, 25]. Awareness and attention to the problem of obesity, but also the “normalizing” of overweight which change people’s perception of their weight status, could have an effect on the way how people respond [2]. Therefore, for the next HIS, measuring height and weight in a random subsample could be very useful in order to assess and apply new correction factors to the whole population.

## Conclusions

Through the national Food Consumption Survey (FCS) 2014, the bias of the self-reported BMI related to the measured BMI could be assessed in Belgium. Based on these data, a simple correction factor (measured BMI/self-reported BMI) was estimated. Applying this correction factor on the self-reported BMI of the national Health Interview Survey (HIS) 2013 led to a more accurate estimation of the obesity prevalence, which is important for decision making. Therefore regular adjustment of self-reported obesity estimates is recommended.

## Abbreviations

BMI: Body mass index
CI: Confidence interval
FCS: Belgian National Food Consumption Survey
HIS: Belgian National Health Interview Survey
